# Decreases in the sustained firing capacity of layer 2/3 pyramidal neurons in the anterior cingulate cortex of aged rats

**DOI:** 10.3389/fnagi.2026.1824113

**Published:** 2026-05-11

**Authors:** Taketoshi Sugimura, Yasuhiko Saito

**Affiliations:** Department of Neurophysiology, Nara Medical University, Kashihara, Nara, Japan

**Keywords:** aging, anterior cingulate cortex, intrinsic electrophysiological properties, patch-clamp recording, sodium channel inactivation, sustained firing, whole-cell recording

## Abstract

The anterior cingulate cortex (ACC) is particularly vulnerable to aging, which impairs cognitive functions such as attention and working memory. Although aging is known to alter intrinsic electrophysiological properties in other brain regions, the differences in the properties of aged ACC neurons and young adult neurons remain uninvestigated. In this study, we compared the intrinsic membrane properties and firing characteristics of aged layer 2/3 pyramidal neurons (26-month-old rats) with those of young adult neurons (6-month-old rats) using whole-cell patch-clamp recordings in acute slices from male Long-Evans rats. Although the distribution of the aged neurons based on the firing patterns did not significantly differ from that of the young adult neurons, the aged neurons exhibited depolarized resting membrane potentials, decreased input capacitance, and increased input resistance. In regular-spiking neurons, compared with young adult neurons, aged ACC neurons presented higher action potential thresholds, smaller action potential amplitudes, narrower action potential half-widths, and smaller slow afterhyperpolarization (sAHP) amplitudes. While basic excitability under standard step currents was preserved, aged regular-spiking neurons did not show sustained firing under ramp or triangular current stimulation, with rapid decreases in the firing frequency following the ramp peak and premature spike termination during the triangular descending phase, respectively. These results indicate that the intrinsic membrane properties of aged ACC neurons differ from those of young adult neurons and that aged neurons exhibit a reduced capacity for sustained firing. This cellular dysfunction in aged neurons provides a potential physiological mechanism underlying the age-related decline in ACC-dependent cognitive functions.

## Introduction

The cognitive decline associated with aging has become a pressing public health concern in modern super-aging societies. This decline results from widespread structural and functional changes in the brain, including decreased volume, synaptic loss, and altered neurotransmission (Fjell and Walhovd, 2010; Morrison and Baxter, 2012; Sikora et al., 2021; Lee and Kim, 2022). However, these changes are not uniform; association cortices, especially the prefrontal cortex (PFC) and its anterior cingulate cortex (ACC), are particularly vulnerable to age-related decline (Raz and Rodrigue, 2006; Pardo et al., 2007; Fjell and Walhovd, 2010; Morrison and Baxter, 2012; Gutchess, 2014).

The ACC is a critical hub for higher-order cognitive functions; this region plays a key role in attention, decision-making, and performance monitoring and contributes to pain processing and behavioral regulation (Carter et al., 1998; Bliss et al., 2016; Anastasiades and Carter, 2021; Le Merre et al., 2021; Howland et al., 2022). In rodents, the ACC is considered homologous to Brodmann area 24 in primates on the basis of anatomical, developmental, and functional evidence (Ongür and Price, 2000; Vogt and Paxinos, 2014; Laubach et al., 2018; Francis-Oliveira et al., 2022; Vertes et al., 2024). It is classified as an agranular cortex lacking a distinct layer 4, where pyramidal neurons in layers 2/3 and 5 play essential roles in intracortical and cortico-subcortical information processing (Harris and Shepherd, 2015; Anastasiades and Carter, 2021; Howland et al., 2022; Vertes et al., 2024). Importantly, the ACC exhibits sustained neuronal activity during attention, working memory, and reward prediction tasks across species (Petit et al., 1998; Monosov, 2017; Wu et al., 2017).

At the cellular level, aging is known to alter the action potential waveform, input resistance, firing frequency, and afterhyperpolarization currents in regions such as the hippocampus and neocortex (Chang et al., 2005; Disterhoft and Oh, 2007; Luebke and Chang, 2007; Luebke and Amatrudo, 2012; Coskren et al., 2015; Rizzo et al., 2015; Severin et al., 2020; Brahimi et al., 2023). Consequently, alterations in the intrinsic electrophysiological properties of neurons are considered critical factors underlying the functional decline of these circuits. However, the specific electrophysiological differences between young adult and aged ACC neurons have not yet been investigated. Therefore, the present study aimed to elucidate the electrophysiological differences in layer 2/3 pyramidal neurons in the ACC of young adult and aged rats. Using whole-cell patch-clamp recordings of acute brain slices, we systematically compared the passive membrane properties, action potential characteristics, neuronal excitability, and dynamic responses to ramp and triangular current stimulation between young adult (6-month-old) and aged (26-month-old) rats. We focused on the rostral aspect of cingulate cortex area 1 (Cg1) and area 2 (Cg2), which correspond to the rodent ACC (Paxinos and Watson, 2007, 2018). These findings provide fundamental insights into cellular alterations in the ACC during aging and suggest a cellular basis for ACC-dependent cognitive decline.

## Materials and methods

All the experimental procedures were approved by the Animal Care Committee of Nara Medical University. The experiments were performed in accordance with the guidelines outlined by the US National Institutes of Health regarding the care and use of animals for experimental research (ARRIVE guidelines 2.0) (Percie du Sert et al., 2020). Fifteen male Long-Evans rats were used in this study (young adult group: 6 months old, *n* = 7; aged group: 26 months old, *n* = 8).

### Slice preparation and whole-cell recording

**Figure 1:**
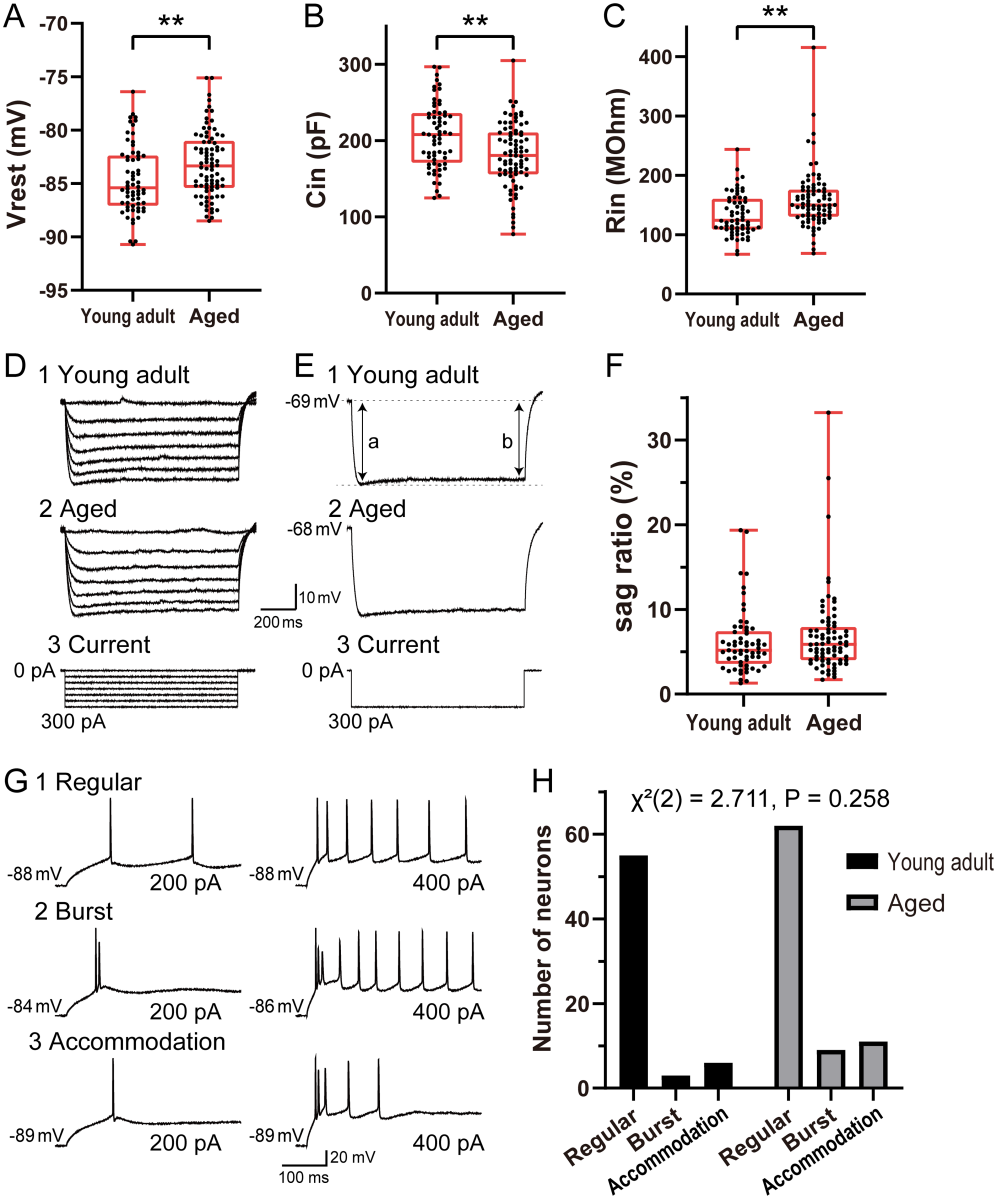
Comparison of the intrinsic membrane properties and firing patterns of ACC layer 2/3 pyramidal neurons from young adult (6-month-old) and aged (26-month-old) rats. **(A–C)** Distributions of the resting membrane potential **(A)**, input capacitance **(B)**, and input resistance **(C)** between young adult (*n* = 64) and aged (*n* = 82) ACC neurons. **(D)** Representative voltage responses of young adult **(1)** and aged **(2)** ACC neurons to hyperpolarizing current pulses **(3**, 1 s duration, −50 pA steps**)**. **(E)** Voltage responses of the same neurons **(D1,D2)** to a − 300 pA current pulse **(3)**. **(F)** Comparison of the sag ratio between young adult (*n* = 61) and aged (*n* = 76) ACC neurons. **(G)** Examples of three firing patterns observed for ACC neurons: regular-spiking, burst-spiking, and accommodation-spiking. For each pattern, the voltage traces in response to two injected currents are shown. **(H)** Distribution of the firing pattern types among all ACC layer 2/3 pyramidal neurons recorded in this study (young adult: *n* = 64; aged: *n* = 82). The distribution of firing patterns did not differ significantly between the groups [*χ*^2^(2) = 2.711, *p* = 0.258, chi-square test]. Data are presented as box-and-whisker plots indicating the median and interquartile range (IQR); the whiskers represent the minimum and maximum values, and the data points represent individual neurons in **(A–C)** and **(F)**. *n* indicates the number of neurons analyzed. The asterisks indicate a significant difference between groups (* *p* < 0.05; ** *p* < 0.01).

The slice preparation and whole-cell patch-clamp recording procedures were similar to those previously described (Saito and Yanagawa, 2017; Sugimura et al., 2025). Briefly, under deep isoflurane anesthesia, a rat was decapitated, and its brain was quickly removed. Frontal brain slices (250 μm thick) that included the cingulate cortex were cut using a microslicer (Pro 7, Dosaka EM, Kyoto, Japan) in an ice-cold modified extracellular sucrose solution containing (in mM) 234 sucrose, 2.5 KCl, 1.25 NaH_2_PO_4_, 10 MgSO_4_, 0.5 CaCl_2_, 26 NaHCO_3_, and 11 glucose, bubbled with 95% O_2_ and 5% CO_2_. Although the definition of the ACC regions remains controversial, Cg1 (area 24b) and Cg2 (area 24a) are consistently regarded as components of the ACC under both traditional and revised classifications (Paxinos and Watson, 2007, 2018). Therefore, in the present study, slices were prepared from rostral ACC regions defined as Cg1 and Cg2 according to the atlas of Paxinos and Watson (approximately +2.28 to −0.36 mm from the bregma). The slices were recovered in an interface-type chamber perfused with an extracellular solution containing (in mM) 125 NaCl, 2.5 KCl, 2 CaCl_2_, 1 MgCl_2_, 1.25 NaH_2_PO_4_, 26 NaHCO_3_, and 25 glucose and aerated with 95% O_2_ and 5% CO_2_ (pH 7.4) at 33 °C for 1 h; thereafter, the slices were incubated in oxygenated extracellular solution at room temperature. For the recordings, each slice in a submerged recording chamber was continuously perfused with extracellular solution at 3 mL/min. The bath temperature was maintained at 30–32 °C using an in-line heater (SH-27A; Warner Instruments, Hamden, CT). In both the young adult and aged rats, pyramidal neurons were readily distinguished from interneurons on the basis of their large soma and prominent apical dendrites. Accordingly, whole-cell current-clamp and voltage-clamp recordings were obtained from these visually identified neurons in layer 2/3 of the ACC using an infrared differential interference contrast optics system (BX51WI; Olympus, Tokyo, Japan) with a 60 × 1.00 NA water-immersion lens. A MultiClamp 700B amplifier (Molecular Devices, Sunnyvale, CA) was used, and the data were acquired using a pCLAMP 10 system (Molecular Devices, Sunnyvale, CA). Patch pipettes were filled with an internal solution containing (in mM) 120 K-methyl sulfate, 10 KCl, 0.2 EGTA, 2 MgATP, 0.3 NaGTP, 10 HEPES, 10 Na_2_-phosphocreatine, and 0.1 spermine, and the pH of the solution was adjusted to 7.3 with KOH. The intracellular solution contained 0.1 mM spermine to prevent the washout of endogenous polyamines, thereby maintaining the physiological rectification properties of inward rectifier potassium channels. This composition follows established electrophysiological protocols (e.g., Saito and Yanagawa, 2017; Sugimura et al., 2025). The osmolarity of the internal solution ranged from 280 to 290 mosmol/L, and the resistance of the patch electrodes was 3–7 MΩ in the bath solution. The voltage and current signals were subjected to low-pass filtering at 3 kHz and digitized at 10 kHz. This rate provides a temporal resolution of 100 μs, which was sufficient for accurately capturing action potential frequency and basic waveform parameters, such as half-width (IQRs for both age groups were between 1.1 and 1.5 ms). To ensure consistency and avoid recording artifacts, waveform analysis was performed using the built-in detection algorithms in the software without additional interpolation or manual smoothing of the raw data. All membrane potentials were corrected for a − 5 mV liquid junction potential during analysis. Bridge balance was applied to compensate for voltage errors due to series resistance during the current-clamp recordings. The resting membrane potential was recorded immediately upon rupture of the cell membrane. During the current-clamp recordings, the membrane potentials of the neurons were maintained between −75 and −65 mV via a continuous constant current injection, and depolarizing and hyperpolarizing current pulses (1 s in duration) were subsequently applied. Current-clamp sweeps were delivered at intervals of 4–5 s. The rheobase current was defined as the minimum depolarizing current (incremented in 10 pA steps) that elicited a single action potential during a 1-s current injection. To classify the firing patterns (Figure 1G), depolarizing current pulses (1 s in duration) were applied from the resting membrane potential. For ramp current stimulation, depolarizing ramp currents were injected for 2 s, reaching final amplitudes of 100–400 pA (corresponding to slopes of 50–200 pA/s), followed by a constant current step at the final amplitude for an additional 2 s. Additionally, triangular ramp currents were applied, consisting of a 2-s ascending phase followed by a 2-s descending phase, with the peak amplitudes ranging from 100 to 600 pA (corresponding to slopes of 50–300 pA/s). For the ramp and triangular current stimulations, recordings in which the first action potential occurred approximately 0.5–1.0 s and approximately 0.5 s after the onset of the ascending phase, respectively, were analyzed.

### Data analysis

**Figure 2:**
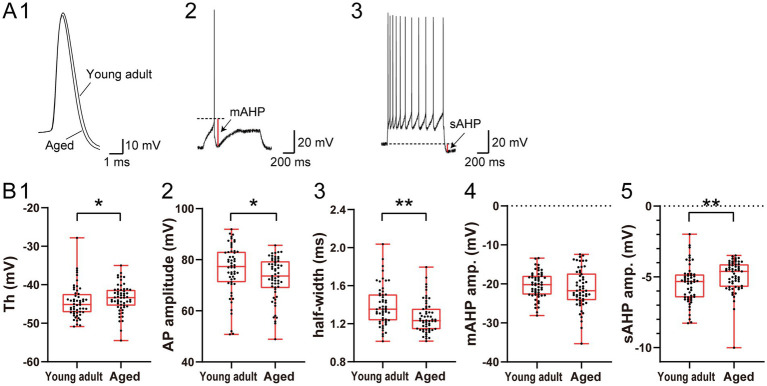
Comparison of the action potential and afterhyperpolarization properties between young adult and aged ACC layer 2/3 regular-spiking pyramidal neurons. **(A)** Representative traces from young adult and aged ACC regular-spiking neurons. **(1)** Superimposed traces of single action potentials evoked by a rheobase depolarizing current step. **(2)** Current-clamp trace illustrating the medium afterhyperpolarization (mAHP) measured at the rheobase current. The dashed line indicates the action potential threshold. **(3)** Trace illustrating slow afterhyperpolarization (sAHP) following a train of action potentials elicited by a 1-s depolarizing current step at the rheobase current + 300 pA. The dashed line indicates the baseline potential. **(B)** Comparisons of the action potential and afterhyperpolarization properties between young adult (*n* = 54) and aged (*n* = 57) regular-spiking neurons: **(1)** threshold, **(2)** amplitude, **(3)** half-width, **(4)** mAHP amplitude, and **(5)** sAHP amplitude. The data points represent individual neurons. The asterisks indicate a significant difference between groups (* *p* < 0.05; ** *p* < 0.01).

**Figure 3:**
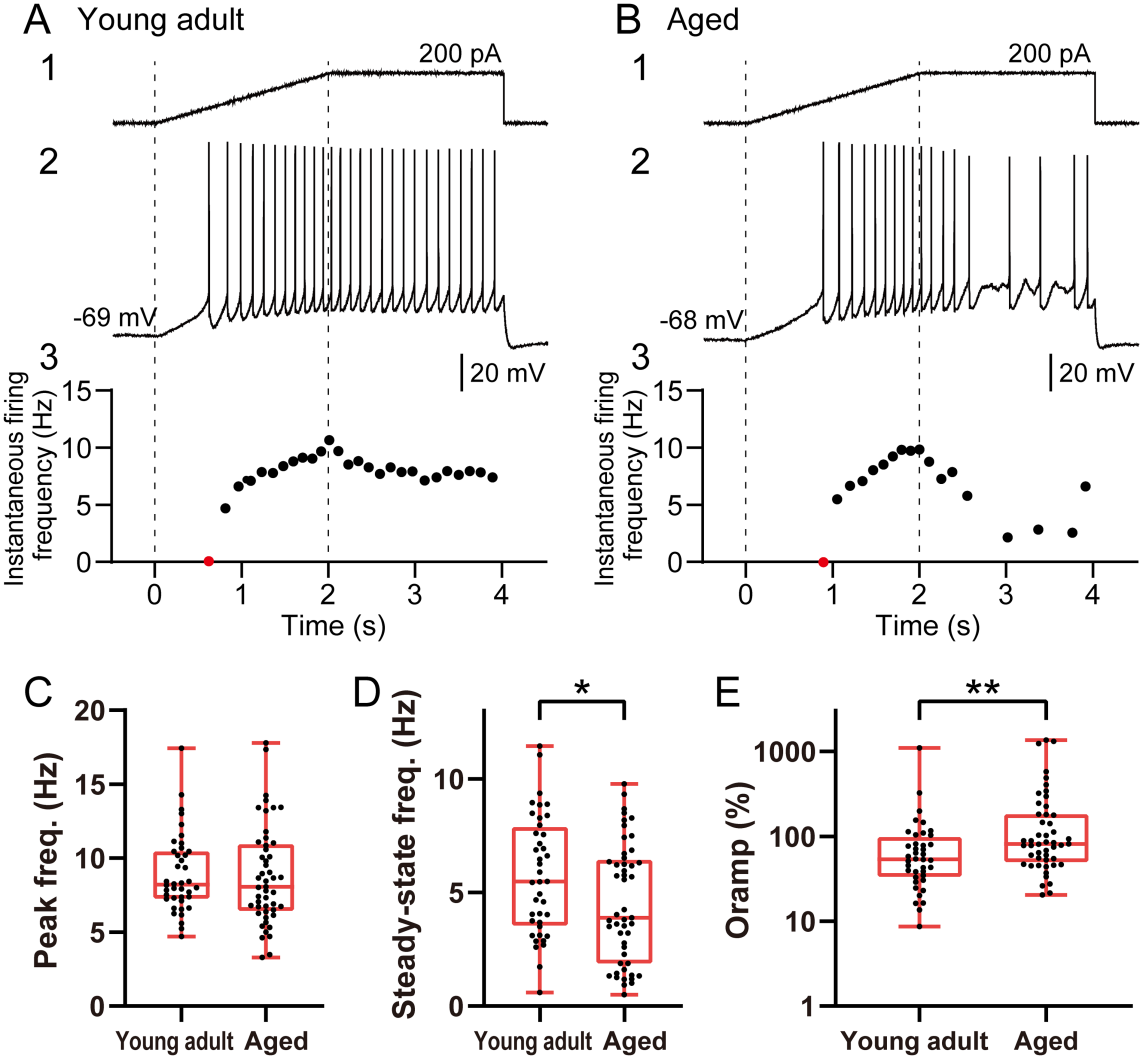
Comparison of the firing responses to ramp current stimulation between young adult and aged ACC layer 2/3 regular-spiking pyramidal neurons. **(A,B)** Representative firing responses to ramp current stimulation from young adult and aged ACC layer 2/3 regular-spiking pyramidal neurons. **(1)** Injected current (0–2 s ramp, 2–4 s steady state). **(2)** Firing responses. **(3)** Time course of the instantaneous firing frequency during ramp stimulation. The first spike on the ascending ramp cannot be calculated as the reciprocal of the ISI; thus, it is plotted on the X-axis and highlighted in red. **(C–E)** Comparisons between young adult (*n* = 40) and aged (*n* = 49) regular-spiking neurons: **(C)** peak firing frequency, calculated as the mean instantaneous firing frequency during 1.8–2.0 s of ramp stimulation, **(D)** steady-state firing frequency, calculated as the mean instantaneous firing frequency during 3.0–4.0 s of the steady-state phase, and **(E)** overshooting firing index (Oramp), which reflects the relative reduction in the firing rate from the peak to the steady state for individual neurons (a higher Oramp value indicates a greater reduction in firing). The data points represent individual neurons. Note that the Y-axis in **(E)** is shown on a logarithmic scale. The asterisks indicate a significant difference between groups (* *p* < 0.05; ** *p* < 0.01).

**Figure 4:**
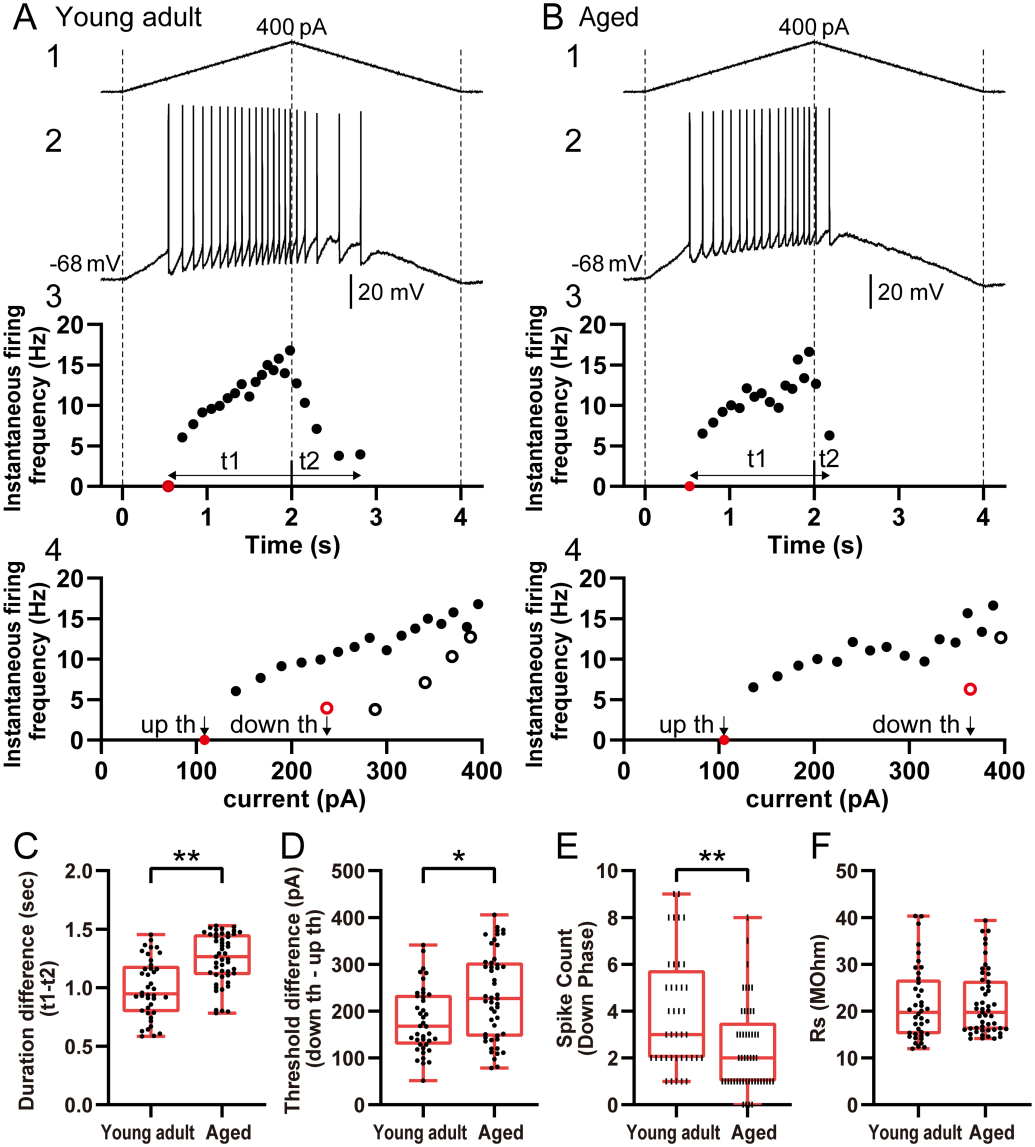
Reduced firing responses to triangular ramp stimulation in aged ACC layer 2/3 regular-spiking pyramidal neurons. **(A,B)** Representative firing responses of ACC layer 2/3 regular-spiking pyramidal neurons from young adult and aged rats during triangular ramp stimulation (2-s ascending and 2-s descending). The traces show the **(1)** injected current, **(2)** firing response, **(3)** instantaneous firing frequency over time (*t*₁ and *t*₂ indicate the firing periods during the ascending and descending ramps, respectively), and **(4)** the relationship between instantaneous firing frequency and injected current. The closed and open symbols represent the ascending and descending ramps, respectively. In **(3)** and **(4)**, the first spike on the ascending ramp is plotted on the X-axis and highlighted in red because it cannot be calculated as the reciprocal of the ISI. In **(4)**, the last spike on the descending ramp is also shown in red to indicate the current threshold for spike termination (down th). **(C,D)** Comparisons of the **(C)** duration difference (*t*₁–*t*₂; difference in the firing duration between the ascending and descending ramps) and **(D)** threshold difference (down th – up th; difference in the injected current thresholds for spike initiation and termination) between young adult (*n* = 40) and aged (*n* = 49) regular-spiking neurons. **(E–F)** Graphs showing **(E)** the spike count during the descending phase (*t*₂) of triangular ramp stimulation and **(F)** series resistance (Rs). Each point represents an individual neuron. The neurons analyzed in Figure 4 are the same as those analyzed in Figure 3. The asterisks indicate a significant difference between groups (* *p* < 0.05; ** *p* < 0.01).

**Figure 5:**
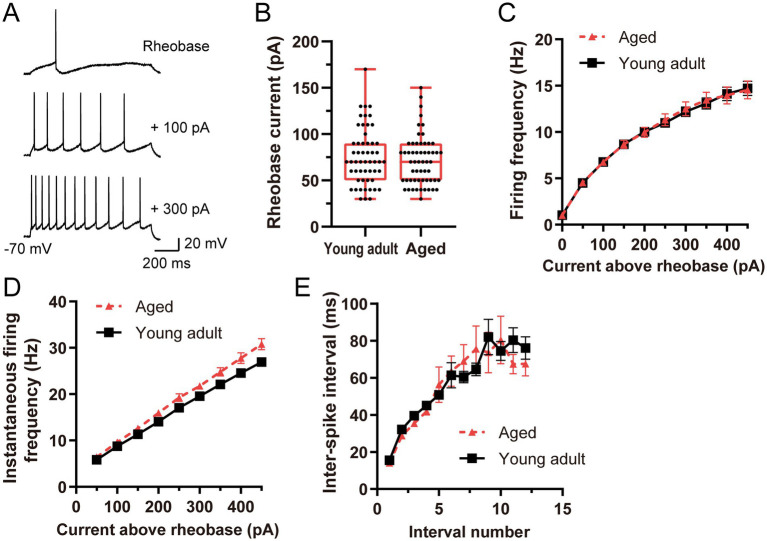
Firing responses of ACC layer 2/3 regular-spiking pyramidal neurons to depolarizing current steps in young adult and aged rats. **(A)** Representative voltage traces during 1-s depolarizing pulses at the rheobase current, rheobase current + 100 pA, and rheobase current + 300 pA. **(B)** Comparison of the rheobase current between young adult (*n* = 54) and aged (*n* = 57) regular-spiking neurons. The data are presented as box-and-whisker plots, with the individual data points representing single neurons. **(C)** Firing frequency–current (f–I) relationship. Current steps were applied from the rheobase current to the rheobase current + 450 pA in 50 pA increments. The data are plotted as the mean firing frequency, with the error bars indicating the SEM. **(D)** Instantaneous firing frequency (IFF)–current relationship. The data are plotted as the mean IFF, with the error bars indicating the SEM. **(E)** Interspike interval (ISI) transitions plotted against the interval number during a 1-s depolarizing current step at the rheobase current + 400 pA. The data are plotted as the mean ISI, with the error bars indicating the SEM.

Offline analysis was performed using AxoGraph X software (RRID: SCR_014284). Neurons with a resting membrane potential below −65 mV and action potential peaks above 0 mV were selected for further analysis. Neurons were classified into three types on the basis of the firing characteristics described in previous studies (McCormick et al., 1985; Connors and Gutnick, 1990; Dégenètais et al., 2002). Regular-spiking neurons were characterized by spike frequency adaptation, where the interspike intervals progressively increased during depolarizing current injections. Burst-spiking neurons were characterized by an initial transient burst of two or more spikes accompanied by an after depolarization (ADP) at the time of the first action potential elicited from the resting membrane potential. Accommodation-spiking neurons, corresponding to fast-adapting neurons (Dégenètais et al., 2002), showed a pronounced increase in the ISI, leading to early termination of the spike train. The input capacitance was determined on the basis of the current induced by a 5-mV voltage step from a holding potential of −70 mV using the membrane test protocol in pCLAMP. The input resistance was estimated on the basis of the voltage response induced by a hyperpolarizing current pulse of −50 pA. The series resistance (Rs) under voltage-clamp conditions was estimated using Ohm’s law on the basis of the difference between the baseline current at −70 mV and the peak inward current during a 10 mV step to −80 mV. The height of the action potential was defined as the difference between the peak of the action potential and the action potential threshold, which was the membrane potential at which the derivative of the voltage trace reached 10 V/s. The half-width of the action potential was defined as the spike width of the half-amplitude from the threshold. The amplitude of the medium AHP (mAHP), defined as the difference in voltage between the most negative point of the AHP and the action potential threshold, was quantified following a single action potential induced by the rheobase current (Figure 2A). The amplitude of the slow AHP (sAHP) was measured from the baseline membrane potential during the prepulse period to the most negative potential occurring after a train of action potentials evoked by a 1-s depolarizing current step at the rheobase current plus 300 pA (Figure 2A). The sag ratio was calculated on the basis of the voltage response to −300 pA current pulses, expressed as the ratio of the sag amplitude (a–b) to the peak voltage deflection (a); this value is presented as a percentage (Figure 1E). The instantaneous firing frequency (IFF) was calculated as the reciprocal of the interspike interval (ISI). For the analysis of firing responses to 1-s depolarizing current steps, the mean IFF was calculated by averaging the reciprocals of all ISIs within the 1-s step for each current intensity. ISI transitions were assessed by measuring consecutive ISIs sequentially and plotting them as a function of the interval number (e.g., 1st, 2nd, 3rd interval, etc.) during a 1-s depolarizing current step at the rheobase current + 400 pA. To quantify the overshooting firing index (Oramp) (Saito and Yanagawa, 2017) (Figure 3E), the peak firing frequency and steady-state firing frequency were defined as the mean instantaneous firing frequency during 1.8–2.0 s and 3.0–4.0 s of the ramp stimulation, respectively. Oramp was calculated using the formula: Oramp = (Peak − Steady-state)/Steady-state × 100%. In the analysis of the firing responses to triangular ramp current stimulation, the difference in the firing duration between the ascending and descending phases was calculated as *t*₁–*t*₂ (Saito and Yanagawa, 2017) (Figure 4C). Here, *t*₁ represents the firing duration during the ascending phase (measured from the first spike to the phase end at 2 s), and *t*₂ represents the duration during the descending phase (measured from the phase starting at 2 s to the last spike) (Figures 4A3,B3). Additionally, the threshold difference was calculated as down th–up th (Figure 4D), where the thresholds for spike initiation (up th) and termination (down th) were defined as the injected currents at which the first and last action potentials occurred, respectively (Figures 4A4,B4). Sample sizes for Figures 3, 4 are smaller than for Figure 5 because only an identical subset of neurons meeting three criteria was analyzed: (1) spikes in both peak (1.8–2.0 s) and steady-state (3.0–4.0 s) ramp intervals for Oramp calculation; (2) first-spike latency 0.5–1.0 s after ramp onset; and (3) first-spike latency ~0.5 s after triangular ascending phase onset. These criteria ensured standardized dynamics and consistent comparison across protocols within the same neurons.

Numerical values are presented as medians with interquartile ranges (IQRs) because most datasets did not satisfy the assumption of normality. Accordingly, the data in the box-and-whisker plots show the median and IQR, with the whiskers extending to the minimum and maximum values and the individual data points representing single neurons. In contrast, the firing frequency–current (f–I) relationships (Figure 5) are plotted as the mean firing frequency with the standard error of the mean (SEM). In all the cases, *n* indicates the number of neurons analyzed. Statistical analyses and figure generation were performed using GraphPad Prism 8 (GraphPad Software). Normality was evaluated using the Shapiro–Wilk test. Because most parameters violated normality assumptions, all two-group comparisons were performed using the nonparametric Mann–Whitney U test. For the f–I and IFF–I relationships and interspike interval (ISI) transitions, the data were log-transformed [Y = log10(Y)] to better approximate a normal distribution. Data without missing values were analyzed using two-way repeated-measures ANOVA, followed by Bonferroni’s multiple comparisons test. For datasets containing missing values (e.g., due to premature firing termination at high-intensity current steps), two-way mixed-effects ANOVA followed by Bonferroni’s multiple comparisons test was employed. For both ANOVA models, Geisser–Greenhouse correction was applied to account for potential violations of sphericity. Statistical significance was defined as *p* < 0.05.

## Results

### Comparison of the intrinsic membrane properties and firing-pattern distributions of young adult and aged neurons

To determine how aging affects the intrinsic membrane properties of ACC layer 2/3 pyramidal neurons, we compared passive electrophysiological parameters between young adult (6-month-old rats, *n* = 64) and aged (26-month-old rats, *n* = 82) ACC neurons using whole-cell patch-clamp recordings. The resting membrane potential was significantly more depolarized for aged neurons than for young adult neurons (−83.4 [−85.4 to −81.0] mV vs. −85.4 [−87.1 to −82.4] mV; *p* = 0.005; Figure 1A). The input capacitance was also significantly lower for aged neurons than for young adult neurons (180.6 [156.2–210.6] pF vs. 207.8 [171.2–235.9] pF; *p* = 0.001; Figure 1B). Moreover, the input resistance was significantly greater for aged neurons than for young adult neurons (150.0 [130.6–175.6] MΩ vs. 124.1 [109.0–160.2] MΩ; *p* < 0.001; Figure 1C). These results indicate that aging induces alterations in the passive membrane properties of ACC neurons.

Because hyperpolarized-activated cyclic nucleotide-gated (HCN) channels regulate intrinsic properties such as the resting membrane potential and input resistance (Nishitani et al., 2019), we assessed their potential involvement by analyzing voltage responses to hyperpolarizing current pulses. In ACC neurons from both the young adult group (*n* = 61) and the aged group (*n* = 76), the degree of voltage sag—characterized by rapid hyperpolarization followed by slow depolarization—was small (Figures 1D,E). Quantitative analysis of the sag ratio revealed median values of 5.2 [3.6–7.4] in the young adult group and 5.9 [4.0–7.9] in the aged group, with no significant difference between groups (*p* = 0.264; Figure 1F). These findings indicate that voltage sag does not differ significantly with age, suggesting similar HCN channel involvement in the two groups.

To assess whether aging affects the diversity of the firing patterns of ACC layer 2/3 pyramidal neurons, we classified all the young adult (*n* = 64) and aged (*n* = 82) ACC neurons recorded in this study into three categories on the basis of their firing behavior: regular-spiking, burst-spiking, and accommodation-spiking neurons (Figure 1G). Among the neurons from young adult rats, 55 were regular-spiking neurons, 3 were burst-spiking neurons, and 6 were accommodation-spiking neurons, whereas the neurons from aged rats included 62 regular-spiking, 9 burst-spiking, and 11 accommodation-spiking neurons (Figure 1H). To assess differences in the distribution of the firing patterns between the two age groups, we performed a chi-square test [*χ*^2^(2) = 2.711, *p* = 0.258], which revealed no significant differences between the two groups. These findings indicate that aging does not significantly affect the diversity of the firing patterns of ACC neurons. While the passive membrane properties shown in Figures 1A–C,F represent pooled data from all identified subtypes, the specific properties for each subtype are summarized in Supplementary Table 1. Except for regular-spiking neurons, aging did not significantly affect the resting membrane potential, input resistance, or input capacitance in the other subtypes. Given that regular-spiking neurons constituted the predominant population in both groups, we focused our subsequent analyses exclusively on this subtype to assess changes in the electrophysiological properties of aged neurons without the confounding effects of cell-type heterogeneity.

### Differences in the action potential waveform and afterhyperpolarization properties between young adult and aged neurons

To clarify differences in the action potential waveform and afterhyperpolarization properties between young adult and aged neurons, we analyzed single spikes evoked by rheobase current and afterhyperpolarization following spike trains in regular-spiking neurons (young adult: *n* = 54; aged: *n* = 57) (Figure 2A). The sample size differs from that of the analysis shown in Figure 1 because this analysis was restricted to regular-spiking neurons in which action potentials were successfully elicited by rheobase current stimulation. Compared with that of the young adult neurons, the threshold for spike initiation was significantly more depolarized for the aged neurons (−43.4 mV vs. −45.1 mV, *p* = 0.048; Figure 2B1). The amplitude of the single action potentials was also significantly smaller for aged neurons than for young adult neurons (73.6 mV vs. 77.3 mV, *p* = 0.023; Figure 2B2). Furthermore, compared with that of the young adult neurons, the half-width of the action potentials of the aged neurons was markedly reduced (1.23 ms vs. 1.35 ms, *p* < 0.001, Figure 2B3). With respect to the afterhyperpolarization properties, the amplitude of the medium afterhyperpolarization (mAHP) did not differ substantially between the groups (−21.8 mV vs. −20.2 mV, *p* = 0.284; Figure 2B4). However, the amplitude of the slow afterhyperpolarization (sAHP) following spike trains was significantly smaller for aged neurons than for young adult neurons (−4.6 mV vs. −5.3 mV, *p* = 0.005; Figure 2B5). The action potential and afterhyperpolarization properties for the other two subtypes (accommodation-spiking and burst-spiking neurons) are detailed in Supplementary Table 2. In accommodation-spiking neurons, a significant difference was observed in the spike threshold (*p* = 0.035), whereas no significant differences were found in the other parameters between the groups. Regarding burst-spiking neurons, statistical comparisons were not performed because of the limited sample size in the young adult group (*n* = 2). In summary, compared with young adult regular-spiking neurons, aged regular-spiking neurons exhibited depolarized spike thresholds, reduced action potential amplitudes, shorter action potential half-widths, and smaller sAHP amplitudes.

### Firing responses of young adult and aged neurons under step current stimulation

We next examined whether the observed changes in the intrinsic properties of the neurons affected the excitability of ACC layer 2/3 regular-spiking pyramidal neurons (young adult: *n* = 54; aged: *n* = 57) by analyzing the firing responses of the neurons to 1-s depolarizing current steps applied from rheobase current stimulation in 50-pA increments up to the rheobase current plus 450 pA (Figure 5A). The rheobase current did not differ significantly between the young adult and aged neurons (70 [50–90] pA vs. 70 [50–90] pA, *p* = 0.549; Figure 5B). With respect to the f–I relationship (Figure 5C), two-way repeated-measures ANOVA revealed a significant effect of the injected current (p < 0.001), confirming that the firing frequency increased with increasing current intensity. However, the main effect of age was not significant (*p* = 0.580), indicating that the overall firing rates did not differ between young adult and aged neurons. The interaction effect between age and current was also not significant (*p* = 0.985), indicating that the increase in the firing frequency with increasing current intensity was similar in both groups. Furthermore, we analyzed the firing properties of accommodation-spiking neurons (Supplementary Figure 1). The rheobase current did not differ significantly between the groups (Supplementary Figure 1A; *p* = 0.697). With respect to the f-I relationship (Supplementary Figure 1B), the main effect of age was not significant (*p* = 0.584), and the interaction effect between age and current was also not significant (*p* = 0.277). Burst-spiking neurons were excluded from this analysis because of the limited sample size (*n* = 2). To further investigate the temporal firing dynamics, we analyzed the IFF and ISI transitions (Figures 5D,E and Supplementary Figures 1C,D). In regular-spiking neurons, while aged neurons exhibited subtly but significantly higher instantaneous firing frequencies (main effect of age on IFF, *p* = 0.0275; Figure 5D), their ISI transitions were not significantly altered (main effect of age, *p* = 0.2042; Figure 5E). Similarly, for accommodation-spiking neurons, although a significant interaction effect between age and current intensity was observed for IFF (*p* = 0.0211; Supplementary Figure 1C)—indicating some intensity-dependent differences in firing dynamics—their overall ISI transitions remained stable between the groups (main effect of age, *p* = 0.1691; Supplementary Figure 1D). Overall, these results demonstrate that under 1-s depolarizing current steps, aging does not significantly impair the fundamental firing capacity or input–output relationships of regular-spiking or accommodation-spiking neurons, despite subtle alterations in their instantaneous firing dynamics.

### Firing responses of young adult and aged neurons under ramp current stimulation

To investigate the firing responses to dynamic depolarization, we analyzed the firing responses of regular-spiking neurons (young adult: *n* = 40; aged: *n* = 49) during a 2-s depolarizing ramp current stimulation followed by a 2-s steady-state phase (Figures 3A,B). Both groups of neurons fired during the ramp phase and continued firing into the steady-state phase. The peak firing frequency near the end of the ramp (1.8–2.0 s) did not differ significantly between the two groups (aged: 8.1 [6.4–10.9] Hz vs. young adult: 8.2 [7.3–10.5] Hz; *p* = 0.643; Figure 3C). In contrast, compared with young adult neurons, aged neurons exhibited a significantly lower steady-state instantaneous firing frequency (during 3.0–4.0 s) (3.9 [1.9–6.4] Hz vs. 5.5 [3.6–7.9] Hz, *p* = 0.038; Figure 3D). Consequently, the overshooting firing index (Oramp), which reflects the relative decrease in the firing rate from the peak phase to the steady-state phase for individual neurons, was significantly greater for aged neurons than for young adult neurons (aged: 81.2 [49.8–181.6]% vs. young adult: 53.7 [33.3–97.6]%; *p* = 0.005; Figure 3E). These results indicate that while the peak response to ramping input is preserved during aging, aging specifically impairs the capacity to sustain firing during the subsequent steady depolarization phase.

### Firing responses of young adult and aged neurons under triangular ramp current stimulation

To further assess the dynamic input–output properties and hysteresis of the young adult and aged neurons, we analyzed the responses of regular-spiking neurons (young adult: *n* = 40; aged: *n* = 49) to triangular ramp current stimulation (2-s ascending phase followed by a 2-s descending phase; Figures 4A,B). Both groups of neurons fired during the ascending phase, and the firing rates of both types of neurons decreased in the descending phase. Notably, compared with young adult neurons, aged neurons stopped firing earlier in the descending phase. Quantitative analysis revealed that the difference in the firing duration between the ascending and descending phases (*t*₁–*t*₂; Figures 4A3,B3) was significantly greater for aged neurons (aged: 1.27 [1.11–1.46] s; *p* < 0.001; Figure 4C) than for young adult neurons (young adult: 0.95 [0.79–1.18] s). Furthermore, the difference in the injected current thresholds for spike initiation and termination (termed the threshold difference, calculated as down th – up th; Figures 4A4,B4) was significantly greater for aged neurons (aged: 227.0 [145.6–303.8] pA; *p* = 0.010; Figure 4D) than for young adult neurons (young adult: 167.7 [128.6–234.5] pA). Consistent with the early cessation of firing, the spike count during the descending phase was lower for aged neurons (aged: 2 [1–3.5] spikes) than for young adult neurons (young adult: 3 [2–5.75] spikes; *p* = 0.001; Figure 4E). The series resistance measured immediately after the firing response was recorded under triangular ramp current stimulation did not differ between the two groups (aged: 19.8 [16.0–26.5] MΩ vs. young adult: 19.7 [15.1–26.7] MΩ; *p* = 0.619; Figure 4F), ruling out the recording quality as a factor impacting the results. Combined with the results of the ramp stimulation experiments, these findings demonstrate that aging significantly alters the dynamic response properties of ACC layer 2/3 regular-spiking pyramidal neurons, specifically impairing their ability to sustain firing during prolonged or fluctuating depolarization periods.

## Discussion

The persistent activity of ACC neurons is known to support a wide range of higher-order cognitive functions, including sustained attention, delay-period maintenance in working memory tasks, encoding of reward uncertainty, and pain anticipation (Petit et al., 1998; Monosov, 2017; Wu et al., 2017; Urien et al., 2018). In these prior studies, *in vivo* unit recordings across ACC neurons and human fMRI data have been used to demonstrate that ACC activity and neuronal firing are significantly elevated above baseline levels during stimulus anticipation and memory retention periods, reflecting the maintenance of cognitive states. Conversely, reduced persistent firing has been reported to correlate with poorer attention maintenance, diminished pain anticipation, and altered processing of reward/punishment uncertainty (Monosov, 2017; Wu et al., 2017; Urien et al., 2018). Moreover, aging has been shown to impair cognitive performance in rats, as evidenced by poor outcomes in sustained attention tasks (Harati et al., 2011), working memory tasks (Bernaud et al., 2021), and cognitive flexibility tasks involving rule switching (Bizon et al., 2012; Brockett and Roesch, 2021). In our *in vitro* analyses, compared with young adult neurons, aged ACC neurons exhibited a rapid decrease in the firing rate after the peak under ramp current stimulation and terminated spiking prematurely during the descending phase under triangular ramp stimulation. These findings suggest that the cellular mechanism underlying age-related cognitive decline may involve a reduced ability of ACC neurons to sustain firing under dynamic depolarization conditions. The distribution of the firing pattern types, with the neurons classified as regular-spiking, burst-spiking, or accommodation-spiking, did not differ significantly across the two age groups, indicating that aging does not alter the overall composition of the firing patterns but rather impairs the ability to maintain firing rates among each type of neuron. This premature termination of spiking is particularly significant, as the inability to sustain firing at the cellular level may destabilize the reverberating circuit between the ACC and the mediodorsal thalamic nucleus (MD), which is implicated in sustaining ACC activity (Rikhye et al., 2018). Consequently, our findings suggest that the instability in the sustained firing of aged neurons at the cellular level may result in circuit-level dysfunction, which may contribute to deficits in attention and working memory.

Termination during repetitive firing has been reported for both hippocampal and neocortical pyramidal neurons (Fleidervish et al., 1996; Upchurch et al., 2022). According to previous hippocampal and neocortical studies, this termination may be attributed to long-term inactivation (LTI; Jung et al., 1997; Mickus et al., 1999; Dover et al., 2010; Upchurch et al., 2022) or slow inactivation (SI; Fleidervish et al., 1996; Vilin and Ruben, 2001; Ulbricht, 2005; Silva, 2014; Chen et al., 2024) of voltage-gated Na^+^ channels following repetitive firing or prolonged depolarization. These inactive states of Na^+^ channels persist for a long time and require hundreds of milliseconds to several seconds for recovery. Because the termination of firing observed for the ACC neurons in this study was quite similar to the observations for hippocampal and neocortical pyramidal neurons, the termination of the firing of ACC neurons may be attributed to Na^+^ channel inactivation. Compared with young adult neurons, aged ACC neurons terminated firing significantly earlier during the descending phase of triangular ramp stimulation. The early termination of the sustained firing of aged ACC neurons may be caused by age-related differences in the properties and distribution of Na^+^ channels. In addition, Na^+^ channels in aged ACC neurons may easily become inactivated. This idea is supported by the higher input resistance of these neurons, which promotes Na^+^ channel inactivation in aged neurons (Figure 1C), although the increase in the input resistance can increase excitability when the number of available Na^+^ channels is reduced, as indicated by the decrease in the action potential amplitude (Figure 2B2). The observed increase in input resistance and the concurrent decrease in input capacitance in aged neurons may reflect a reduction in the total membrane surface area, which is consistent with age-associated dendritic regression (Coskren et al., 2015). The increase in input resistance resulting from these structural changes likely leads to more pronounced and prolonged membrane depolarization during sustained stimulation. This sustained depolarization, in turn, promotes the entry of Na + channels into SI or LTI states (Fleidervish et al., 1996; Mickus et al., 1999; Upchurch et al., 2022). Furthermore, the slow AHP (sAHP) amplitude of aged neurons was reduced (Figure 2B5). Because recovery from SI and LTI states is promoted by membrane hyperpolarization (Fleidervish et al., 1996; Jung et al., 1997; Dover et al., 2010), the sAHP facilitates the recovery of inactivated Na^+^ channels by providing sustained hyperpolarization during repetitive firing. For pyramidal neurons, while the medium AHP (mAHP) is mediated by apamin-sensitive small-conductance Ca^2+^-activated K^+^ (SK) currents, the sAHP is mediated by apamin-insensitive Ca^2+^-dependent K^+^ currents (Sah and Faber, 2002; Bond et al., 2004). The absence of significant differences in the mAHP between young adult and aged neurons suggests that SK channel functions are preserved during aging, while the apamin-insensitive sAHP is reduced only in aged neurons. These differences in the input resistance and sAHP may increase the susceptibility of aged neurons to LTI and SI compared with young adult neurons. Therefore, the impairment in sustained firing in the aged ACC should be viewed as a complex interplay between biological structural alterations that increase susceptibility to depolarization and the resulting ion channel dysfunction. However, importantly, without direct voltage-clamp measurements of Na + currents, this structural–functional link remains a proposed model.

In addition to intrinsic membrane properties, metabolic factors may critically contribute to impaired sustained firing in aged neurons *in vivo*. Maintaining repetitive firing imposes a heavy bioenergetic burden, as a substantial portion of cellular energy is consumed by Na^+^/K^+^-ATPase to restore ionic gradients (Attwell and Laughlin, 2001). Aging is widely associated with mitochondrial dysfunction and altered energy metabolism (Grimm and Eckert, 2017; Mattson and Arumugam, 2018), which can limit the ATP supply required for ionic pump activity during prolonged stimulation. In the present *in vitro* study, however, neurons were dialyzed with an internal solution containing sufficient ATP and phosphocreatine to buffer energy demands. The failure of aged neurons to maintain sustained firing under these energy-replete conditions suggests that alterations in intrinsic electrophysiological properties, such as Na+ channel inactivation, rather than metabolic failure, are the primary drivers of the firing deficits observed in vitro. Under physiological conditions in vivo, where neurons must rely solely on their own aged mitochondria, the cumulative ATP deficit during prolonged activity may further exacerbate this intrinsic membrane instability, leading to more pronounced firing failures.

In the present study, we identified pyramidal neurons based on characteristic morphological features under IR-DIC microscopy. Although we focused our analyses on the predominant regular-spiking class, importantly, layer 2/3 regular-spiking pyramidal neurons in the ACC represent a heterogeneous population with distinct projection targets (e.g., local versus long-range projections) and molecular profiles. As shown in Figure 3D, the steady-state firing frequencies in aged neurons exhibited a broad distribution that appeared to form a bimodal distribution. This pattern could be due to certain subpopulations being particularly susceptible to aging. If age-related vulnerability is indeed subtype specific, the observed changes in the RS population would reflect a composite of resilient and affected cells rather than a uniform shift across all neurons. This implies that our reported median might underestimate the actual degree of impairment within the most vulnerable subpopulations. However, because *post hoc* anatomical or molecular identification was not performed and because this apparent separation was not consistently observed across all dynamic protocols (e.g., Figure 4), we exercised caution and did not further subdivide these neurons for the current analysis. Identifying the specific factors that contribute to such cellular-level differences remains important to address in future studies to understand the fundamental diversity and mechanisms of brain aging. In this study, 250 μm slices were utilized to facilitate the visualization of neurons in the opaque aged brain. Although this thickness is thinner than the standard 300 μm preparation, the improved tissue transparency in our setup allowed us to target neurons located deeper from the slice surface, thereby minimizing the influence of surface truncation. The absence of age-related differences in input resistance and input capacitance within the accommodation-spiking subtype (Supplementary Table 1), which also comprises large pyramidal neurons (Dégenètais et al., 2002), indicates that the 250 μm thickness did not introduce systematic truncation bias or survival bias in aged animals. Therefore, the robust changes observed specifically in regular-spiking neurons primarily reflect intrinsic biological aging rather than slicing artifacts. All experiments in this study were conducted using male rats. Given the evidence of sex differences in neuronal physiology and aging processes, the exclusive use of males represents a limitation of our study. Future research including female animals is necessary to determine if our findings represent a generalized feature of brain aging.

Finally, while our study focused on changes in the properties of aged layer 2/3 pyramidal neurons in the ACC, the anatomical definition of the ACC is critical for interpreting our results. In earlier anatomical frameworks (Paxinos and Watson, 2007), the ACC was defined by Cg1 and Cg2, excluding areas A25 (infralimbic cortex) and A32 (prelimbic cortex). More recent classifications (Paxinos and Watson, 2018) have expanded the ACC to include A25, A32, and the rostral portions of Cg1 and Cg2 (corresponding to A24a and A24b), whereas the caudal portions of Cg1 and Cg2 have been reassigned to the midcingulate cortex (MCC). Efforts to harmonize nomenclature between rodents and primates have led to ongoing debates regarding ACC boundaries (van Heukelum et al., 2020; Anastasiades and Carter, 2021; Francis-Oliveira et al., 2022). To accommodate both frameworks, we targeted the rostral portions of Cg1 and Cg2 (corresponding to A24a and A24b in the updated nomenclature), which are consistently recognized as part of the ACC across different anatomical frameworks. Therefore, the impairment in sustained firing of the aged ACC neurons observed in our study suggests a functional decline in the ACC.

## Data Availability

The raw data supporting the conclusions of this article will be made available by the authors, without undue reservation.
