# Supplementary material for: Decreases in the sustained firing capacity of layer 2/3 pyramidal neurons in the anterior cingulate cortex of aged rats
Source: Front Aging Neurosci. 2026 May 11;18:1824113. doi: 10.3389/fnagi.2026.1824113 (PMC13199164; doi:10.3389/fnagi.2026.1824113)
Supplement: Supplementary file 1 [file Data_Sheet_1.docx]

**Decreases in the sustained firing capacity of layer 2/3 pyramidal neurons in the anterior cingulate cortex of aged rats**

Running title: Reduced firing in aged ACC

**Taketoshi Sugimura^1*^ and Yasuhiko Saito^1^**

^1^ Department of Neurophysiology, Nara Medical University, Kashihara, Nara 634-8521, Japan.


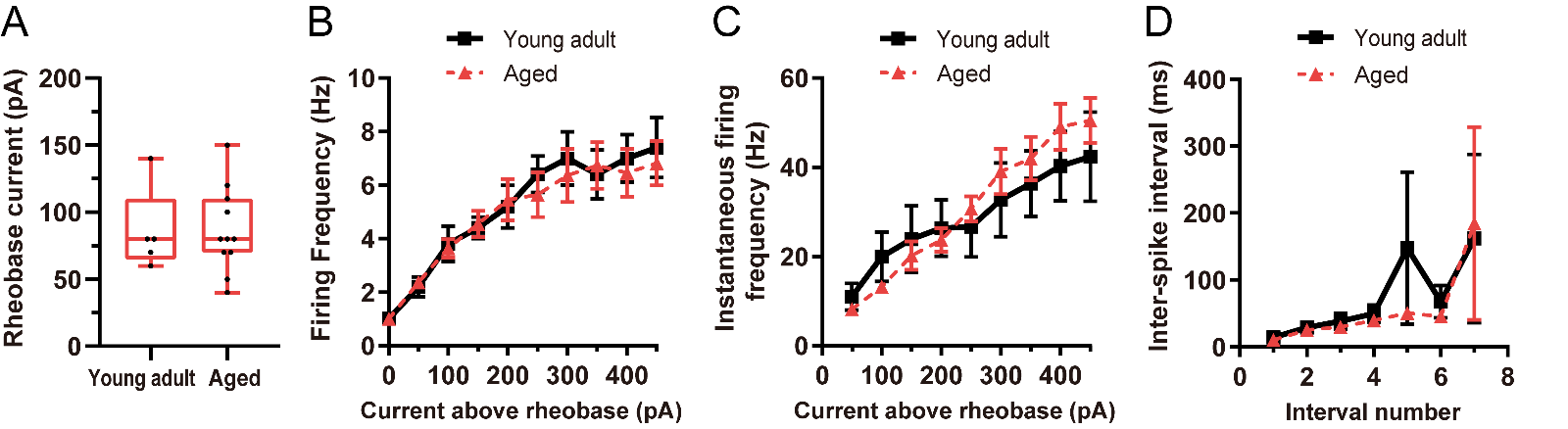


**Supplementary Figure 1. Firing responses of ACC layer 2/3 accommodation-spiking pyramidal neurons to depolarizing current steps in young adult rats and aged rats.**

(A) Comparison of the rheobase current between young adult and aged accommodation-spiking neurons. The data are presented as box-and-whisker plots, with the individual data points representing single neurons. (B) Firing frequency–current (f–I) relationship. Current steps were applied from the rheobase current to the rheobase current + 450 pA in 50 pA increments. The data are plotted as the mean firing frequency, with the error bars indicating the SEM. (C) Instantaneous firing frequency (IFF)–current relationship. The data are plotted as the mean IFF, with the error bars indicating the SEM. (D) Interspike interval (ISI) transitions plotted against the interval number during a 1-s depolarizing current step at the rheobase current + 400 pA. The data are plotted as the mean ISI, with the error bars indicating the SEM.

| **Supplementary Table 1. Passive membrane properties of ACC pyramidal neuron subtypes.** | | | |
| --- | --- | --- | --- |
|  |  |  |  |
| Subtype / Parameter | Young adult (n) | Aged (n) | P-value |
| **Regular-spiking** |  |  |  |
| Resting membrane potential (mV) | −85.7 [−87.2, −82.7] (55) | −83.9 [−86.2, −81.2] (62) | 0.004** |
| Input Capacitance (pF) | 210.7 [183.8, 237.1] (55) | 187.9 [161.7, 213.5] (62) | 0.001** |
| Input Resistance (MΩ) | 123.2 [108.6, 153.8] (55) | 148.2 [128.8, 169.3] (62) | < 0.001** |
| Sag ratio (%) | 5.1 [3.4, 6.4] (54) | 5.3 [4.0, 6.9] (57) | 0.645 |
|  |  |  |  |
| **Accommodation-spiking** |  |  |  |
| Resting membrane potential (mV) | −81.0 [−84.8, −78.0] (6) | −83.4 [−85.1, −79.8] (11) | 0.462 |
| Input Capacitance (pF) | 163.0 [131.5, 202.0] (6) | 179.4 [160.4, 206.3] (11) | 0.591 |
| Input Resistance (MΩ) | 154.5 [119.0, 186.3] (6) | 149.9 [124.7, 171.7] (11) | 0.808 |
| Sag ratio (%) | 7.3 [3.7, 11.4] (5) | 6.8 [3.6, 9.8] (11) | > 0.999 |
|  |  |  |  |
| **Burst-spiking** |  |  |  |
| Resting membrane potential (mV) | −78.8 [−84.3, −78.8] (3) | −82.3 [−83.2, −80.0] (9) | 0.577 |
| Input Capacitance (pF) | 160.5 [156.9, 178.6] (3) | 129.2 [92.9, 169.8] (9) | 0.209 |
| Input Resistance (MΩ) | 163.0 [95.8, 173.3] (3) | 219.2 [165.4, 286.2] (9) | 0.1 |
| Sag ratio (%) | 19.2, 19.3 (2) | 10.7 [9.0, 22.0] (8) | N/A |

Data are presented as the median [interquartile range] (IQR). The number of neurons analyzed is indicated in parentheses (n). Statistical comparisons between the young adult and aged groups were performed using the Mann‒Whitney U test. ** P<0.01. Statistical comparisons were not performed for the sag ratio of burst-spiking neurons because of the limited sample size in the young adult group (n=2), indicated as N/A. Individual values are shown for n=2.

| **Supplementary Table 2. Action potential and afterhyperpolarization properties of ACC pyramidal neuron subtypes.** | | | |
| --- | --- | --- | --- |
|  |  |  |  |
| Subtype / Parameter | Young adult (n) | Aged (n) | P-value |
| **Accommodation-spiking** |  |  |  |
| Threshold (mV) | −41.0 [−44.8, −36.4] (5) | −45.6 [−47.4, −44.7] (11) | 0.035* |
| Amplitude (mV) | 66.4 [60.7, 69.6] (5) | 72.5 [69.9, 79.9] (11) | 0.090 |
| Half-width (ms) | 1.35 [1.09, 1.76] (5) | 1.33 [1.18, 1.49] (11) | 0.827 |
| mAHP amplitude (mV) | −20.7 [−23.1, −15.7] (5) | −16.1 [−20.9, −12.2] (11) | 0.180 |
| sAHP amplitude (mV) | −5.1 [−7.6, −5.0] (5) | −4.7 [−6.0, −3.1] (11) | 0.377 |
|  |  |  |  |
| **Burst-spiking** |  |  |  |
| Threshold (mV) | −43.5, −38.8 (2) | −46.6 [−51.6, −44.1] (8) | N/A |
| Amplitude (mV) | 58.1, 63.9 (2) | 71.3 [63.1, 77.9] (8) | N/A |
| Half-width (ms) | 1.13, 1.31 (2) | 1.28 [1.14, 1.39] (8) | N/A |
| mAHP amplitude (mV) | −23.0, −13.2 (2) | −14.6 [−17.4, −11.6] (8) | N/A |
| sAHP amplitude (mV) | −9.5, −7.6 (2) | −8.2 [−9.8, −7.6] (8) | N/A |

Data are presented as the median [interquartile range] (IQR). The number of neurons analyzed is indicated in parentheses (n). Statistical comparisons between the young adult and aged groups were performed using the Mann‒Whitney U test. * P<0.05. Statistical comparisons were not performed for burst-spiking neurons because of the limited sample size in the young adult group (n=2), indicated as N/A. Individual values are shown for n=2. Abbreviations: mAHP, medium afterhyperpolarization; sAHP, slow afterhyperpolarization.
